# Multi-Mission Earth Observation Data Processing System

**DOI:** 10.3390/s19183831

**Published:** 2019-09-04

**Authors:** Paidamwoyo Mhangara, Willard Mapurisa

**Affiliations:** Earth Observation Directorate, South African National Space Agency (SANSA), The Enterprise Building, Mark Shuttleworth Street, Pretoria 0002, South Africa

**Keywords:** multi-mission, earth observation, data processing, satellite images, remote sensing

## Abstract

The surge in the number of earth observation satellites being launched worldwide is placing significant pressure on the satellite-direct ground receiving stations that are responsible for systematic data acquisition, processing, archiving, and dissemination of earth observation data. Growth in the number of satellite sensors has a bearing on the ground segment payload data processing systems due to the complexity, volume, and variety of the data emanating from the different sensors. In this paper, we have aimed to present a generic, multi-mission, modularized payload data processing system that we are implementing to optimize satellite data processing from historical and current sensors, directly received at the South African National Space Agency’s (SANSA) ground receiving station. We have presented the architectural framework for the multi-mission processing system, which is comprised of five processing modules, i.e., the data ingestion module, a radiometric and geometric processing module, atmospheric correction and Analysis Ready Data (ARD) module, Value Added Products (VAPS) module, and lastly, a packaging and delivery module. Our results indicate that the open architecture, multi-mission processing system, when implemented, eliminated the bottlenecks linked with proprietary mono-mission systems. The customizable architecture enabled us to optimize our processing in line with our hardware capacities, and that resulted in significant gains in large-scale image processing efficiencies. The modularized, multi-mission data processing enabled seamless end-to-end image processing, as demonstrated by the capability of the multi-mission system to execute geometric and radiometric corrections to the extent of making it analysis-ready. The processing workflows were highly scalable and enabled us to generate higher-level thematic information products from the ingestion of raw data.

## 1. Introduction

The systematic data acquisition, processing, archiving, and dissemination of satellite data is a fundamental task performed by most satellite ground receiving stations across the world [1,2,3,4,5]. These ground segment activities are critical in ensuring data utilization and directly impact the success of satellite missions. Core ground segment functions such as data reception, processing, valorization, archiving, discovery, access, and dissemination are evolving with time to meet the increasing demands of modern image processing [1,2,3,4,5,6,7,8]. Direct receiving stations (DRS) receive raw data from the satellite through ground antennas before it is down-converted and demodulated and transferred for systematic processing [6,7,8]. This procedure also involves converting the raw, unitless relative reflectance digital numbers of the original bands into true measures of radiance. Satellite data are increasingly being used to support a wide range of scientific and operational activities related to infrastructure mapping, crop monitoring, water resources assessments, urban monitoring, land use and land cover mapping, natural resources monitoring, and vegetation assessments. There has been a significant rise in the number of satellite launches in the last two decades to meet the ever-increasing demand for medium- and high-resolution earth observation data [9,10,11]. Technological advancements in satellite engineering are increasingly making it feasible to design satellites with very high spatial, spectral, temporal, and radiometric resolutions. These changes are inadvertently exerting significant pressure on the data ground stations charged with processing, archiving, cataloguing, and disseminating these colossal datasets [12]. The variety and sheer size of satellite datasets pose significant operational efficiency challenges to traditional image processing systems.

Remotely sensed data are prone to several systematic and random effects that combine to degrade the quality of satellite imagery received on the ground by DRS. Image restoration procedures are therefore required to rectify these degradation effects. In most cases, ground receiving stations focus their attention on two fundamental image pre-processing procedures, known as geometric and radiometric rectification [13,14,15,16]. Radiometric rectification seeks to eliminate or minimize distortions in the values recorded for image pixels due to atmospheric effects, striping, random noise, and scan line dropouts. Atmospheric haze is known to systematically elevate pixel values due to the preferential scattering of shorter wavelength bands, particularly in the visible region of the electromagnetic spectrum. Random noise normally results from the unpredictable and unsystematic performance of the satellite sensor or data transmission. Image striping is mainly a result of detector calibration problems, while scan line dropouts are caused by signal losses from specific detectors. Geometric rectification aims to register satellite imagery within a specific geographic reference system and to eliminate the systematic distortions resulting from image displacements by applying systematic high order transformations. Three important requirements used in evaluating the geometric performance of satellite systems are absolute geodetic accuracy, band-to-band registration, and image-to-image registration. Image-to-image registration ensures that geometrically corrected images taken at different times are able to be registered to a defined level of accuracy that enables multi-temporal analysis [13,14,15,16].

Radiometric and geometric processing is normally done by sensor-specific product generation systems that pre-process that data to various processing levels. For instance, the Landsat Missions use the Landsat product generation system (LPGS). In the case of Landsat 7, Level 1 data products are radiometrically and geometrically corrected and the processing level is dependent on the availability of ground control points (GCP), a digital elevation model (DEM), and the payload correction data (PCD) collected by the satellite. The LPGS processes three Level 1 products, and these include precision and terrain correction (L1T), systematic terrain correction (L1Gt), and the systematic correction (L1G) products. L1T products are radiometric and geometrically corrected using GCPs and by integrating a DEM to correct for relief displacements. L1Gt products are systematically corrected to attain radiometric and geometric accuracy and use a DEM to compensate for the terrain displacements. L1G products are systematically, radiometrically, and geometrically corrected using PCD. Landsat 7 Level 1 products are delivered as digital numbers (DN), and can easily be rescaled to spectral radiance or top-of-the-atmosphere (TOA) reflectance. The Landsat 8 Level 1 processing system has similar radiometric and geometric processing protocols. The processing algorithms incorporate ancillary data processing, sensor geometric model creation, sensor LOS generation and projection, input/output space correction grid generation, systematic terrain correction and resampling, geometric model precision correction using GCPs, and terrain-corrected image resampling functionality [17,18,19,20].

The naming conventions for the processing levels differ across sensors. For instance, Sentinel-2 data are systematically processed from Level-0 to Level-1C using the instrument data processing functionality of the payload data ground segment. Level-0 data processing is done to package the raw satellite data, and consolidates them with its ancillary raw data for long-term archiving and forward processing to Level-1A, which decompresses the relevant mission source packets. Sentinel-2 Level-1B data are radiometrically corrected using dark signal, crosstalk, and pixel response non-uniformity corrections. The radiometric rectifications are done on Level-1C also include identification of defective pixels as well as deconvolution and denoising of high spatial resolution bands and binning on low-resolution spectral bands. Sentinel-2 Level-1C processing involves radiometric and geometric rectification that includes high accuracy ortho-rectification and spatial registration on a global reference system. Level-2A processing involves the generation of a scene classification and atmospheric correction to a TOA Level-1C product to generate a bottom-of-atmosphere (BOA)-corrected reflectance orthoimage product [21]. The processing levels for CBER-4 are divided into five processing levels, excluding the raw data from the telemetry data received from the downlink. Level-0 is frame synchronized and decommutated digital data, while Level-1 is radiometrically and geometrically corrected raw data. Level-2 is radiometrically and geometrically corrected using a systematic model with GCPs, whereas Level-3 and Level-4 processing use GCPs and a digital terrain model for terrain parallax correction, respectively. Level-5 processing is focused on thematic classification.

Our assessment of the Landsat 7, Landsat 8, Sentinel-2, and CBERS-4 ground segment data processing systems clearly shows the importance of radiometric and geometric subsystems. We further identified the importance of the process control subsystem for scheduling and monitoring the process workflows, a quality assessment subsystem, and a data management subsystem for data management services for efficient operational image pre-processing in our review. The success of earth observation satellite missions is equally dependent on the remote sensing and geographical information systems (GIS) software that enable users to extract information from satellite imagery and use it for analysis and modeling. The Sentinel missions have several open and free remote sensing software packages on the Sentinel Application Platform (SNAP) that offer visualization and the analytical and processing capability necessary to enable the full utilization of Sentinel data.

The processing of satellite data from multiple payloads using single-sensor approaches is inefficient, cumbersome, and poses significant operational challenges. The architectural designs of traditional payload data processing systems are often rigid and tightly coupled to the extent that they inhibit system integration, adaptability, and scalability. To overcome these constraints, large-scale satellite image processing data centers are increasingly developing modularized, multi-mission open architectural designs to process images from multiple missions. There is an increasing tendency to apply multi-mission, modular open architectural designs that are easily adaptable and share a range of interoperable services and tools. The concept of multi-mission ground data systems is not new. Green [4] presented NASA’s advanced multi-mission operations (AMMOS) multi-mission ground data system and outlined its architectural form and functionality. Using AMMOS as a case study, Barnes [1] provided an in-depth analysis of the development of software architecture. The DLR successfully implemented a generic multi-mission modularized data processing known as a data information management system (DIMS) that has end-to-end processing functionality for acquisition, processing, post-processing, archiving, ordering, and delivery [7,22]. DIMS’s flexible, modular system designs, service components, and workflows allow individual components to efficiently execute processing, ordering, publishing, and delivering tasks [6,8,23,24] presented an automatic multi-mission processing system known as CATENA. Built-up by the DLR, CATENA uses integrated grid computing and was grown for processing large volumes of optical satellite data from different sensors such as Landsat, SPOT, Rapideye, Worldview, and Pleiades. This multipurpose, modularized data processing can be integrated into the DIMS system or can be run as a standalone system, and considers the integration of various processing chains from the different modules.

The increasing demand for remotely-sensed satellite data and earth observation services has seen an upsurge in the number of direct reception satellite at the South African National Space Agency’s (SANSA) ground receiving station at Hartebeeshoek, South Africa. Built in the 1950s, the key functions for the ground receiving station include satellite tracking, telemetry and command, in-orbit testing, charge control, space navigation, and direct response to earth observation satellite imagery. It has a wide range of antenna systems that encompass the L, S, and C, Ext C, X, Ku, DBS, S, and Ka frequency bands, of which seven are full-motion Telemetry, Tracking and Command (TT&C) antennas. The X-band antenna dedicated to earth observation data reception has a wide reception footprint that covers the southern African continent from 3° to 50° south. The station has received satellite data from Landsat missions since 1972. To date, the following sensors have been directly received at the Hartebeeshoek ground station: Landsat 2–5, 7, and 8; ERS-1 SumbandilaSAT; SAC-C; CBERS-2B, CBERS-4 (MUX, WFI, P5M, and P10); SPOT 1–7; and RadarSAT-2 satellites. This number is on an upward trajectory if one considers potential sensors that are going to be received through the Brazil, Russia, India, China and South Africa (BRICS) virtual constellations agreements, African Resource Management constellation, and the Sentinel data collaborations. The processing of data being received from sensors currently, such as Landsat 7, Landsat 8, MODIS Aqua and Terra, SPOT 6/7, CBERS-4, and Radarsat-2 already poses significant processing challenges and efficiency issues. In this paper, we aimed to present a generic, multi-mission, modularized payload data processing system that we are implementing to optimize satellite data processing from historical and current sensors directly received at SANSA’s ground receiving station.

## 2. Current Status

The most significant challenge in receiving and processing data from multiple sensors is the different hardware and software configurations that need to be installed and maintained separately for each mission. This causes operational challenges requiring an additional technical staff complement. In addition to technical staff, hardware lifetime has been greatly reduced by the rate of technological advancements, and this often results in a lack of support for older hardware systems. This usually results in the inability to reprocess any received mission data for some missions if hardware failures are encountered. To mitigate all the above-mentioned issues, organizations are exploring the use of multi-mission processing pipelines that are both sensor- and hardware-independent [1,2,4,25,26]. The goal is to have a single, portable processing codebase that supports decommissioned, current, and future missions.

As mentioned before, radiometric and geometric corrections constitute the bulk of the processing requirements. These corrections are universal to all sensors and can thus be standardized for all multi-mission processing. While differing sensor optics might present challenges in standardizing pre-processing, further downstream processing has reached a maturity level where standardization can be implemented, for example, in the use of the V6S atmospheric correction model for all medium- to high-resolution sensors. 

**Figure 1:**
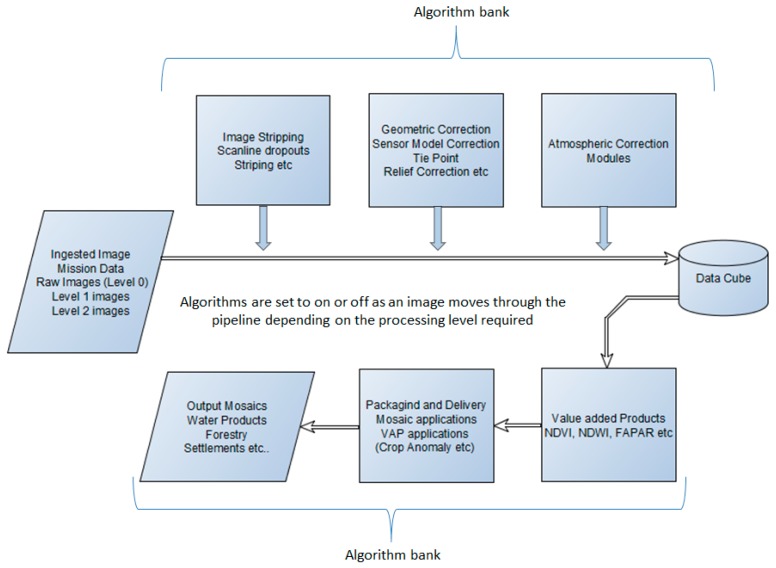
Multi-mission processing concept that is sensor-independent.

The next section discusses the work that has commenced in implementing a multi-mission processing system at SANSA. By implementing generic algorithms for various processing stages, the concept of a multi-mission processing system has been, to a greater extent, realized. Figure 1 below illustrates the multi-mission concept that has been adopted.

The idea behind the multi-mission concept is to develop generic sensor-independent algorithms that are easily adaptable to different sensors, either by providing sensor-specific parameters or using a generic model that is universally accepted for sensors (e.g., V6S models). Each sensor will provide the algorithm with custom parameters that will be used in processing the sensor.

## 3. Implementation Architecture

**Figure 2:**
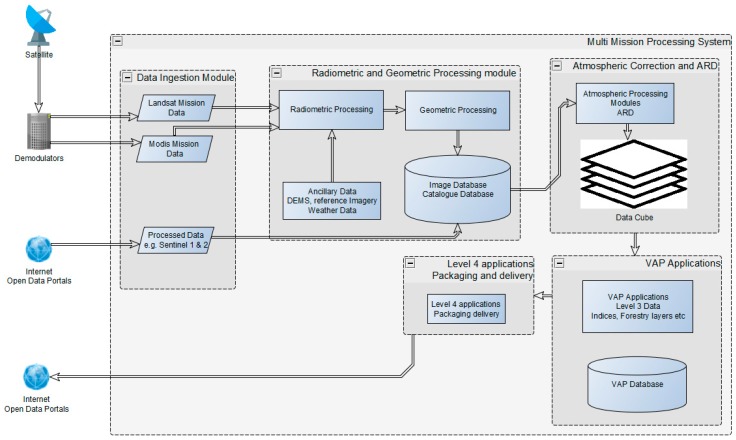
Implementation of the multi-mission processing system, which is comprised of four processing modules.

The framework proposed here does not assume a pre-defined input image level for processing. The system is designed to ingest images at different levels of processing. Depending on the required output, only the required algorithms are triggered as the image moves through the processing pipeline. To allow for multi-sensor storage in a standardized data management system, data cubes are used to allow for further processing using generic algorithms that are non-sensor-specific. The diagram below shows a more detailed framework with all the different modules that constitute the entire system. Figure 2 illustrates the processing framework that has been implemented thus far. The framework is comprised of four modules. Each module is sensor-independent and the task of each is described below.

While there has been a drive to develop multi-mission systems, most of the solutions are proprietary. In addition, due to the emergence of different hardware requirements and evolving technologies and software, the drive has been to develop custom solutions while keeping abreast of technological advancements in high-performance computing. The SANSA multi-mission system (MMS) aims to leverage open source software and libraries in delivering a cost-effective solution. While other solutions exist, the SANSA MMS aims to leverage new automated systems for scaling applications in High-Performance Computing (HPC) environments. The MMS presented here uses a Kubernetes system, which had not been developed when other legacy systems such as DIMS and CATENA were launched and which uses open source and customized software for end to end processing. Further to this, the SANSA MMS integrates architectural concepts such as the data cube that have not yet been reported in DIMS, CATENA, and AMMOS.

### 3.1. Technical Implementation of the Multi-Mission Processing System

The multi-mission data processing system was developed in the C++ programming language with additional programming languages such as Python and JavaScript. On its backend, the data processing system uses my PostgreSQL object-relational databases that are integrated into the PostGIS extension to enhance its spatial intelligence. To optimize processing efficiency, the algorithms were customized for parallel processing using the open multi-processing (OpenMP) Application Programming Interface (API). OpenMP provides a simple and flexible interface necessary for supporting parallel processing applications using shared memory multi-processing running on multiple servers. Deployed on CENTOS 7 server operating systems, the multi-mission data processing system utilizes the open-source Slum Workload Manager for job scheduling and cluster management. 

We utilized the Geospatial Data Abstraction Library (GDAL) generic open-source platform as a mechanism to read and write common satellite image file formats. GDAL is a powerful and widely used translator library for raster and vector geospatial data formats (https://gdal.org/). Our implementation uses GeoNetwork as a catalogue application for the management of geospatial data sources. GeoNetwork provides metadata editing and search functionality and is compliant with ISO metadata standards such as 1SO 19115:2003—Metadata, ISO 19115-2 for Gridded Imagery Extension, ISO 19,110—Feature Catalo, ISO 19,119—Services, and ISO 19115-1-Metadata Fundamentals (https://geonetwork-opensource.org). Current developments involve integration of the generic iCOR atmospheric correction algorithm, which has been proven to work effectively with Landsat-8/OLI and Sentinel-2/MSI data and is independent of scene and sensor [27]. The multi-mission data processing also integrates insight toolkit (ITK) open-source libraries with potential algorithms for registering, segmenting, analyzing, and quantifying satellite data [28]. Provenance collection is an important step in image processing in that it supports satellite data preservation and stewardship. In remote sensing terms, provenance can be regarded as the process of documenting the lineage of the geoprocessing undertaken on the data [29]. Provenance capturing is, therefore, a critical component of complex satellite data processing workflows, in that it automatically captures the sequence of image processing procedures and transformations that were executed to generate products [30,31]. In the multi-mission data processing system, we used the Karma provenance software as a plugin to collect data provenance from the multi-mission data processing workflows. Karma is an open-source software based on the open provenance model (OPM), the modular architecture of which was adaptable to the architectural setup of our multi-mission data processing. Karma has been used successfully by NASA on the Advanced Microwave Scanning Radiometer Earth Observing System [29]. Pérez [32] provides a good review of provenance systems for adoption.

The algorithm bank integrates several cross-platform open-source libraries that are utilized and adopted for image processing. The multi-mission data processing also draws on generic and specialized image processing algorithms available in the Remote Sensing and GIS Library (RSGISLib) [33], which has over 300 algorithms for performing some low-level functions such as stacking image bands, zonal statistics, normalizing data, segmentation, and applying filters. Generic atmospheric and radiometric algorithms available in RSGISLib for multi-mission image processing include the 6S model, Py6s, and the Atmospheric and Radiometric Correction of Satellite Imagery (ARCSI) algorithms. The Orfeo ToolBox (OTB) provides a rich source of algorithms that we integrated into the data processing system, and these include the Haralick Texture and filtering algorithms used for the classification of human settlements.

### 3.2. Framework Modules

#### 3.2.1. Data Ingestion

The data ingestion module has two primary roles. The first role is to ingest data from the demodulators and check whether all ancillary files and data from the sensor have been received in full. All the expected data intervals are checked based on the transmission schedules for the day and the databases are updated. Mission data are then archived with all the accompanying metadata. The second role is to check data received from open data portals that have been processed to a higher level than raw data. The ingestion module checks for the level of processing and tags the dataset metadata as it moves to the next module, which will decide on the processing level required based on the tag. The data ingestion module also updates all the relevant databases with regard to received data.

**Figure 3:**
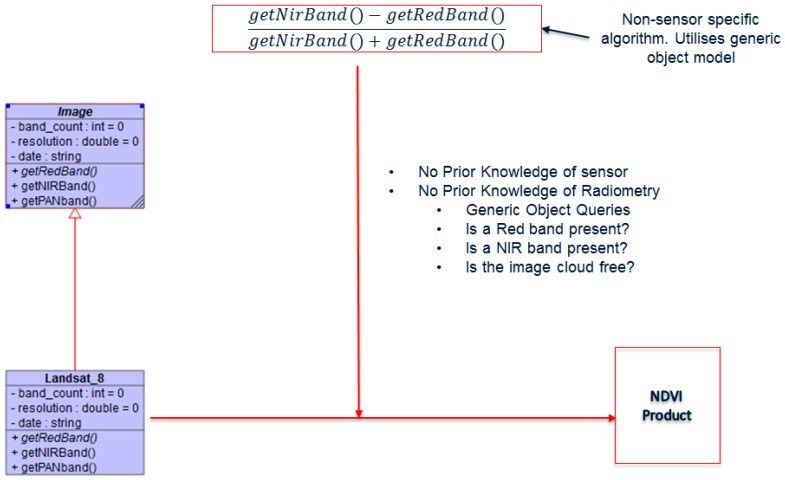
Image object model created at ingest.

A common object model is defined for the sensors, as shown in Figure 3. Each sensor is first converted to this common image object model, which is defined by a generic set of virtual routines that exist for all sensors. Additionally, all sensor data are converted to the TIFF file format at ingestion. Each sensor object then inherits the virtual routines and provides an implementation for that routine if the bands that support the function are present, otherwise the function remains virtual. Downstream processing modules only request data using the generic functions instead of checking what type of sensor is being processed. Figure 3 illustrates the object model that is created for each sensor once ingested. This common object model does not alter original downlinked data, but only created at ingest to allow downstream processing. Input data formats that are supported by the SANSA MMS are restricted to all Geospatial Data Abstraction Library (GDAL)-library-supported image formats. 

**Figure 4:**
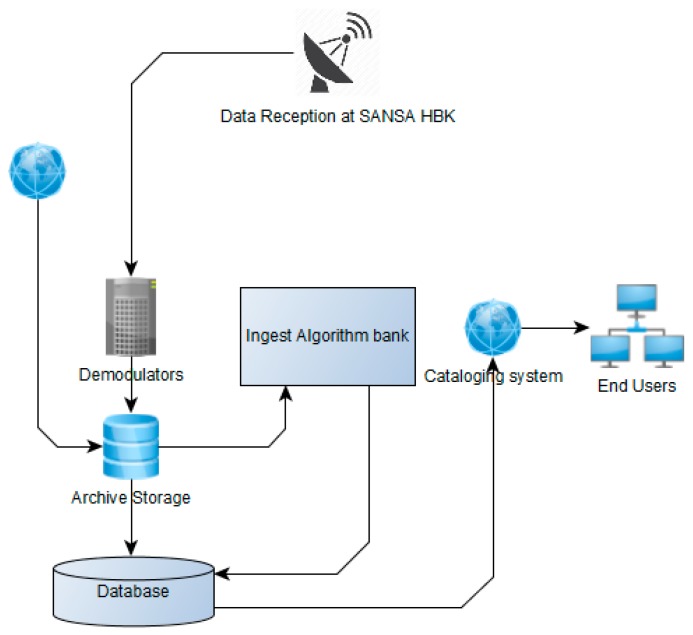
High-level overview of ingest module.

**Figure 5:**
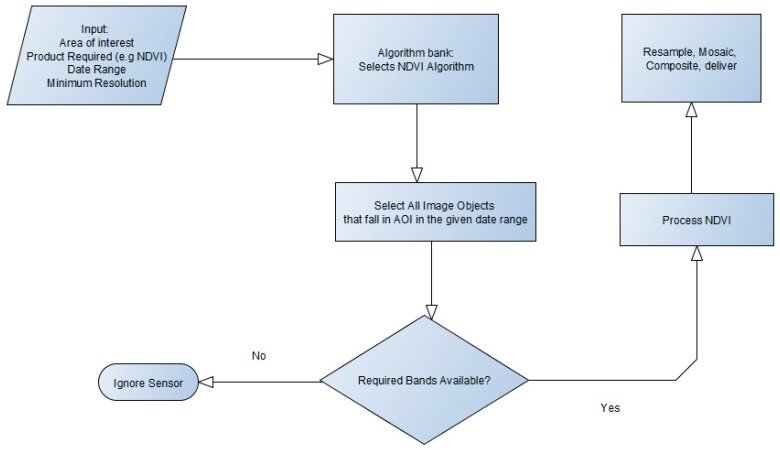
Simplified example of a sensor-independent algorithm that uses an area of interest and bands available at a defined minimum resolution.

The image uses a common image object model to access sensor data. An example of an NDVI query using the mode is shown above. Algorithms request data using a generic function that is customized for each sensor through object inheritance. A high-level overview of the ingest module is shown in Figure 4. Algorithms request for the inputs they require using the generic functions. If the sensor has the requested data within a specified date range, as illustrated in Figure 5, the algorithm executes.

#### 3.2.2. Radiometric and Geometric Processing Module

Radiometric and geometric rectifications are required to correct for errors such as spectral distortions inherent in satellite imagery due to atmospheric, terrain, and relief distortions, sun angle, and satellite drift and positioning. Teillet [15] highlights that radiometric corrections are critical in removing the effects that modify the spectral consistency of land cover features. Radiometric and geometric corrections are particularly important when undertaking change detection studies that involve multi-sensor datasets and time series analysis. In general terms, radiometric correction methods are classified into absolute and relative categories [15,34]. Absolute corrections are aimed at correcting radiance and reflectance using atmospheric models that use onboard sensor calibration data, sun angles, viewing angles, and measured ground data. Relative corrections are aimed at normalizing multi-temporal datasets to given reference data to ensure comparability. Generic geometric rectification is achieved using the generalized rational polynomial coefficients (RPC) sensor model, since it can be utilized without full knowledge of the physical sensor model. For each medium- to high-level sensor that is directly downlinked, RPCs are generated for use in geometric image rectification. The radiometric and geometric module also integrates sensor-specific programs such as the adapted Landsat 8 Level 1 product generation system (LPGS) and SPOT 6 custom developed pre-processing chains for orthorectification, pan sharpening, and mosaicking.

#### 3.2.3. Atmospheric Correction and Analysis Ready Data (ARD) Module

This module receives all the L1 imagery either from the radiometric and geometric processor, or directly from the data ingestion module if the data has already been processed to L1. Atmospheric corrections are then applied to the datasets, resulting in ARD data which are then ingested into a data cube. The data cube serves as a store and data management system for all ARD data that is used to produce value-added products that are non-sensor-specific. Generic algorithms that are implemented are provided by, for example, ESA SNAP or MACCS V6S. The algorithm provided in the software mentioned can be adapted to most sensors. These serve as a basis for atmospheric correction. Since the available atmospheric corrections are limited, the correct model is required for any given sensor. Each sensor object has a sensor ID that stores the original sensor identity. The atmospheric correction model first queries the sensor ID for any image object and uses the appropriate model. 

#### 3.2.4. Value Added Products (VAP) Processing

This module is responsible for producing value-added products from radiometrically and geometrically corrected data. The applications are non-sensor-specific and use data available in the production of any product. Some of the products that are currently in production include waterbody layers and forestry and vegetation products. An application first checks for the availability of data over the area of interest for processing, and uses available data rather than sensor-specific data. Depending on resolution and availability, algorithms request data using coverage only, and not the specific sensor. Various products that have been successfully produced at SANSA can be found in References [35,36,37,38].

The algorithm library consists of a set of stable multi-sensor and non-sensor-specific algorithms used to address a range of operational requirements. Multi-sensor algorithms that have been successfully implemented include techniques for geometric and radiometric rectification, atmospheric correction, image fusion, image mosaicking, vegetation indices, and morphological classification algorithms. For the development of vegetation indices to estimate biomass and vegetation condition, we take advantage of the fact that most vegetation indices are developed from blue, green, red, and near-infrared channels common in the visible and near-infrared (VNIR) spectral region for most satellite sensors. An area of interest is a key requirement when processing a VAP. The workflow shown in Figure 5 below illustrates the requests and queries that are used when processing a VAP without specifying a sensor name. System products are processed in this manner by the MMS. However, ad-hoc user requests that are not systematically processed are handled by the Data Cube platform. 

**Figure 6:**
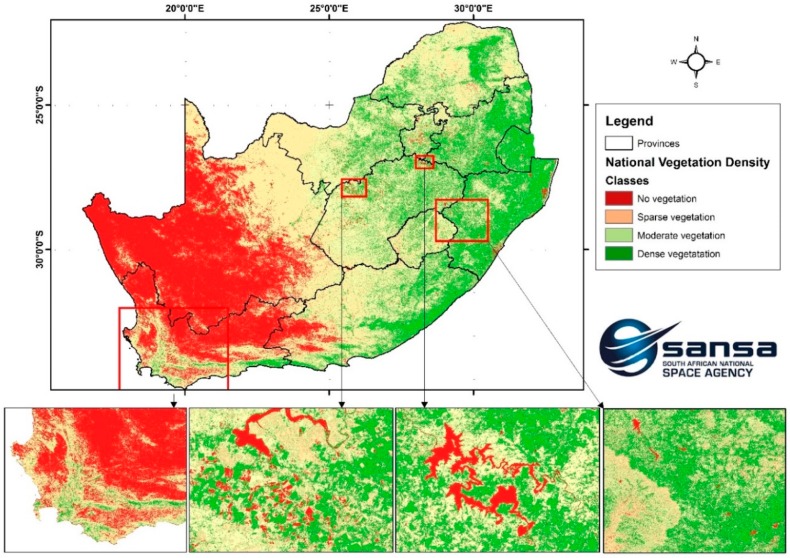
National vegetation density map showing vegetation status for 2018 wet season (Nov–Apr). **Left**: Western Cape’s drier conditions. **Centre-left**: Cropped and fallow areas. **Centre-right**: Vaal Dam water extent. **Right**: Mountain greenness in the Drakensberg Mountains.

**Figure 7:**
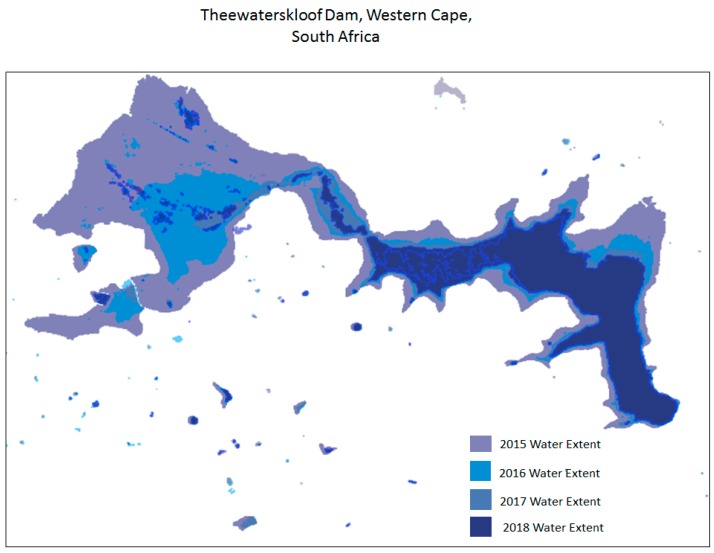
Monitoring of Thewaterskloof Dam in the Western Cape South Africa using time series data.

**Figure 8:**
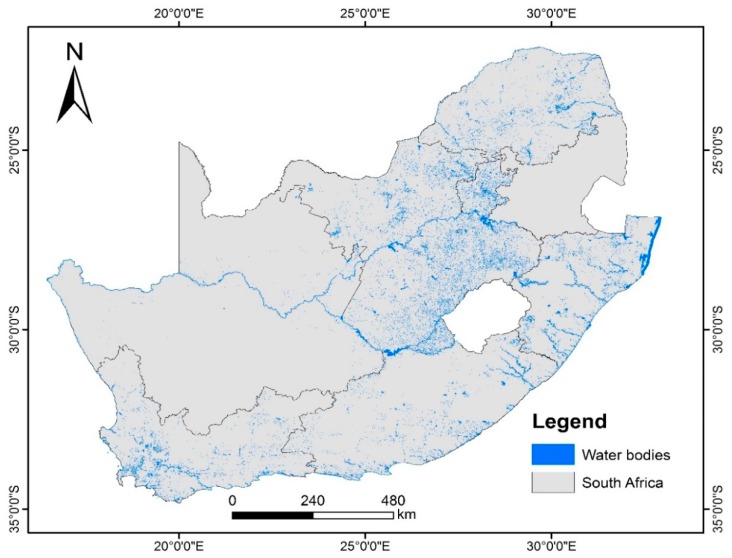
The National Waterbody Layer derived from Landsat 8 images acquired between December 2018 and February 2019.

**Figure 9:**
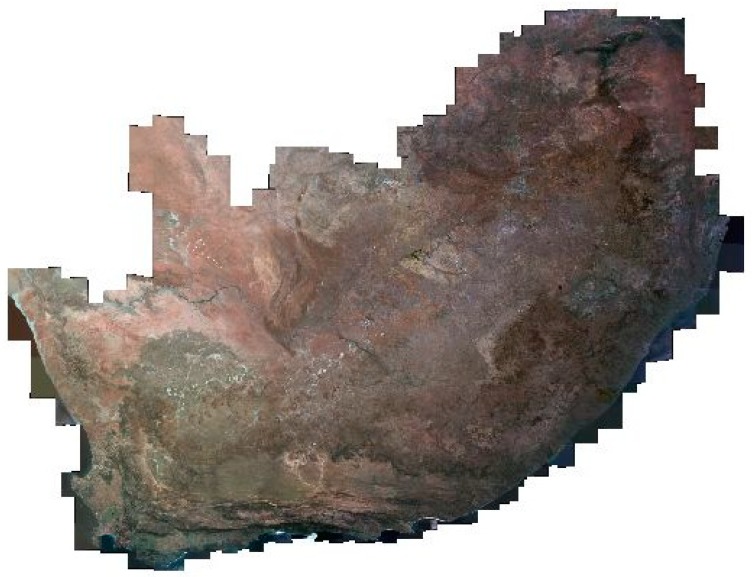
SPOT 6 mosaic of South Africa.

**Figure 10:**
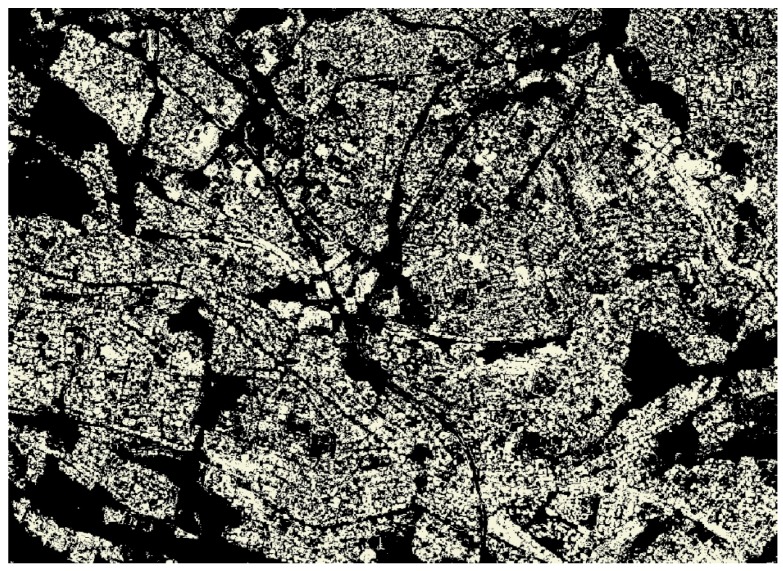
Human settlements layer for Johannesburg, South Africa from SPOT 6.

Our implementation thus embeds common vegetation indices such as the normalized difference vegetation index (NDVI) (Rouse et al. 1974), simple ratio vegetation index (RVI) [39], transformed vegetation index (TVI), and ratio vegetation index [40], which all utilize the red and near-infrared spectral bands. Human settlements layers have been produced using the system. Classification of the human settlements layer is based on sensor-specific adaptations of the morphological and texture-based approaches [41,42,43,44,45,46]. The human settlements classification protocol uses a ruleset that involves the use of Haralick textural features such as Gray level co-occurrence matrices (GLCM) and existing land cover information. Morphological and texture-based classification techniques are increasingly being used for land cover classification due to their stability [47]. The algorithm library currently integrates generic automated land cover classification specifically aimed at extracting vegetation cover, surface water bodies, and human settlements. Vegetation composites are generated using a simple NDVI computation highlighted earlier. Development of generic, stable, and reliable multi-sensor algorithms that are interoperable across a range of satellite sensors is the subject of ongoing research. Examples of some of products that are generated through the multi-mission data processing include the national vegetation density layer (Figure 6), time-series monitoring of surface water bodies (Figure 7), national waterbody layer (Figure 8), SPOT 6 mosaic of South Africa (Figure 9), and the human settlements layer for Johannesburg (Figure 10). The system has also embedded functionality to compute biophysical parameters such as leaf area indices (LAI) and fraction of absorbed photosynthetically active radiation (FAPAR) for Landsat 8 and Sentinel-2 data [48,49,50,51]. Extraction of surface water bodies is currently operationalized on Landsat 8 OLI, using the simple water index to generate surface water bodies shown in Figure 7 and Figure 8. Another simple multi-sensor algorithm for surface water body extraction embedded in the processing system includes the normalized difference water body index (NDWI) proposed by McFeeters [52]. Recently, Rokni [53] confirmed the robustness of the NDWI for mapping lakes using Landsat data. 

#### 3.2.5. Data Cube Module

All processed products either have specific users that require them continuously or need to be discoverable to end-users. This has been achieved through FTP for large data access, a catalogue service for searching and data discovery, and open data sharing that allows for data connection and streaming to the third-party clients. This module prepares processed data so that it can be accessed by any of the means mentioned in this paragraph. 

The implementation of the data cube module is aimed at providing a common analytical framework consisting of a series of data structures and software tools to enable the cataloguing and exploratory data analysis of multiple gridded data collections [54,55,56]. Our design adopts the Open Data Cube architecture and utilizes a variety of open source tools such as the Jupyter Notebooks, Python libraries, Open Data Cube Stats, PostgreSQL database, Open Data Cube Explorer, Command Line Tools, and Open Geospatial Consortium (OGC) web services (www.opendatacube.org).The ODC architecture was selected as it provides a flexible framework for accessing, organizing, querying, and analyzing large quantities of satellite data using open source software that makes customization to the multi-mission faster. As illustrated in the process workflow, the data cube ingests pre-processed data that have been radiometrically and geometrically corrected with atmospheric corrections done to ensure data interoperability. The organization of the data into the data cube is ensured by an indexing process that first defines the products by defining common properties for a collection of data, extracting the metadata, and lastly indexing the data by recording the metadata and its location. The ingestion process is accomplished using ingestion configuration procedures aimed at data reformatting and conversions, e.g., resampling and reprojections. In the short-term, the data cube module is aimed at exploiting analysis ready data (ARD) from the Landsat 8 mission, SPOT 1-6, Sentinel-2, and CBERS-4. The development of this module draws from the experiences of successful data cube implementations such as the Digital Earth Australia and Swiss Data Cube. The technical implementation of the data cube benefits from the technical support of Geoscience Australia, the co-inventors of the data cube. The data cube concepts and lessons learned from developing Digital Earth Australia are presented by Lewis [56]. In the same breath, Giuliani [57] highlighted the experiences of building the Swiss Data Cube. Similarly, Dhu [55] provided some data cube practical applications using Digital Earth Australia that included analysis of flood regimes and groundwater identification. The growing list of countries implementing data cubes provides lessons to guide our implementation; a summary of the status of international data cube developments has been highlighted by Killough [58].

#### 3.2.6. Processing Architecture

**Figure 11:**
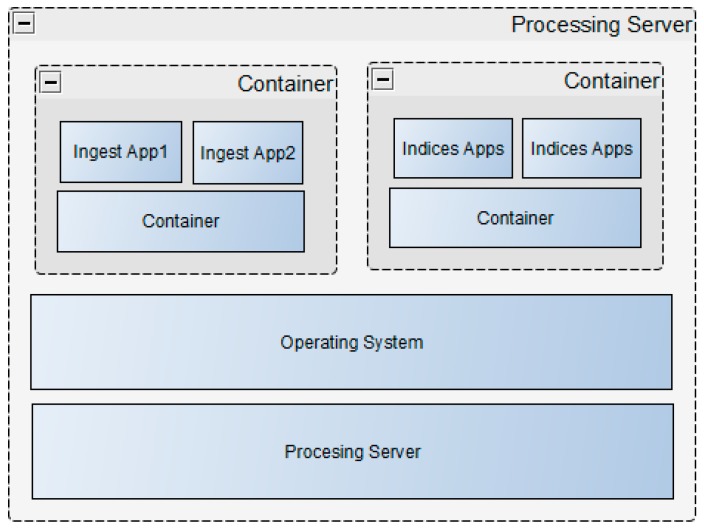
Application deployment, scaling and management are based on Kubernetes. Applications are hosted in containers that can easily be scaled up. Applications also support multi-threads.

The system has two levels of parallelization with regard to processing. The first level is at the application level, where OpenMP uses single application multi-threaded support. This ensures full utilization of available cores on a system by each application when executing a task. The number of cores is requested at run time by each application. In turn, the applications are hosted in containerized environments, which allows for scaling up of the system. The management and scaling of these applications in each module is handled by Kubernetes. Chained applications for each module are hosted in a container. If multiple instances are required for concurrent processing, multiple instances are launched based on available resources. The diagram shown in Figure 11 below illustrates the architecture used in the MMS to support scaling and concurrent processing. 

## 4. Discussion

The implementation of the multi-mission processing system has eliminated the bottlenecks associated with proprietary mono-mission systems and has significantly optimized our large-scale image processing, as demonstrated in this paper. The modularized multi-mission data processing has enabled a seamless end-to-end image processing, as demonstrated by the capability of the multi-mission system to execute geometric and radiometric corrections to the extent of making it analysis-ready. The processing workflows also enabled the generation of higher-level thematic information products from the ingestion of raw data. The multi-mission processing system also takes advantage of open-source image processing algorithms that can easily be adapted and integrated into the common algorithm bank that can be used by a variety of sensors. Proprietary mono-mission data processing systems often have subsystems that are tightly coupled and are closed for integration and adaptation. The modular approach used in the development of the multi-mission processing systems ensures flexibility and easy integration of the processing chains and workflows. 

The ability to customize image processing algorithms has enabled scientists to improve existing algorithms and develop new methodologies that could easily be embedded into the multi-mission processing system. We used the human settlements, surface water body, and vegetation extraction algorithms to demonstrate this capability. The ability to share a common pool of resources, such as reference data and DEMs for geometric rectification, was also valuable. The scalability of the multi-mission data processing has been demonstrated by its adaptability to high-performance computing methods such as parallel processing and multi-threading on single or multiple servers or virtual machines, depending on the system performance requirements. The utility of the multi-mission data processing system as also yielded significant cost savings through the reduction of software licenses associated with proprietary software. The multi-sensor architecture presented in this paper illustrates the benefits of establishing a common architecture for all missions. The flexibility offered by open, modular architectural design enables researchers to develop algorithms that can be integrated into the workflow as plugins upon successful testing. Moreover, multi-mission processing provides some level of harmonization and standardization across sensors. The system is also compliant with future data architectures such as data cubes, and can generate analysis-ready data.

In summary, multi-mission processing systems have various advantages when compared to sensor-dependent systems. A major advantage relates to the cost savings that are realized. Multi-mission processing systems are also hardware-independent, which eliminates the need to have specifically dedicated hardware systems for each sensor. Additionally, the code base for the MMS can easily be adapted to ingest a new sensor, which reduces development time.

The use of the data cube as a platform for data access has various advantages. The data are all processed to analysis-ready data (ARD), which reduces the data preparation burden to end-users. The data are also provided on a common platform that enforces a standard with regards to data indexing and access. This provides end-users with the ability to share algorithms. Furthermore, the provision of a continuous earth data records does not depend entirely on one sensor. The MMS bridges this gap by ensuring continuous data availability on a sensor-independent platform. Time-series applications that require continuous data records can benefit from the gap-filling capabilities that are made possible due to the availability of various sensors. Consequently, the temporal resolution of data is reduced and the impacts from atmospheric effects such as haze and cloud are minimized.

## 5. Conclusions

The ideal goal for the future would be to have a multi-mission processing standard and API that can be shared and adopted by satellite image providers. Customizable and sharable API will alleviate the need to host various hardware and software configurations for ground segments that have multiple data reception licenses. Research and implementation of a product request module from end-users are currently underway. This aims to add extra functionality to the system by allowing user requests for various products.

The presented multi-mission data processing system has several advantages. Firstly, the use of multiple datasets bridges critical spatial and temporal gaps needed in time-series studies. The use of many sensors also improves the revisit times in areas of interest and mitigates the impact of cloud cover. Its modular design also enables the easy integration of higher-level value-added processing algorithms for the production of higher-level products such as NDVI, water body, and human settlements layers, ensuring shorter delivery time to customers. The use of parallel processing, OpenMP, and Kubernetes described earlier enables concurrent processing, scaling, and optimum utilization of computing resources. Moreover, the integration of the data cube enables effective data structuring and cataloguing to support long term environmental studies.

The number of earth observation satellites of which the data are being directly received at the Hartebeeshoek ground receiving station has risen significantly in the last decade, necessitating a multi-mission approach in terms of systematic data processing from ingestion to the generation of higher-level products. In this study, we have presented a functional modularized approach that can be used to systematically pre-process data using generic geometric and radiometric algorithms. Generic atmospheric models such as the 6S models are used to atmospherically correct moderate and high spatial resolution satellite data. The scalable and flexible architecture enables the system to be adaptable when adding new functionality, such as the integration of new algorithms and processors to generate value-added products. The multi-mission approach provides a common architecture for all missions and allows for harmonization and standardization of interfaces. Other advantages of the open multi-mission architecture presented include the ability to plug in processors that are mission-specific, and the re-utilization of mature algorithms and other proven open-source plugins.
